# Canonical Wnt signaling is involved in switching from cell proliferation to myogenic differentiation of mouse myoblast cells

**DOI:** 10.1186/1750-2187-6-12

**Published:** 2011-10-05

**Authors:** Shingo Tanaka, Kumiko Terada, Tsutomu Nohno

**Affiliations:** 1 Department of Molecular and Developmental Biology, Kawasaki Medical School, Kurashiki, Okayama 701-0192, Japan

## Abstract

**Background:**

Wnt/β-catenin signaling is involved in various aspects of skeletal muscle development and regeneration. In addition, Wnt3a and β-catenin are required for muscle-specific gene transcription in embryonic carcinoma cells and satellite-cell proliferation during adult skeletal muscle regeneration. Downstream targets of canonical Wnt signaling are *cyclin D1 *and *c-myc*. However both target genes are suppressed during differentiation of mouse myoblast cells, C2C12. Underlying molecular mechanisms of β-catenin signaling during myogenic differentiation remain unknown.

**Results:**

Using C2C12 cells, we examined intracellular signaling and gene transcription during myoblast proliferation and differentiation. We confirmed that several Wnt signaling components, including *Wnt9a, Sfrp2 *and *porcupine*, were consistently upregulated in differentiating C2C12 cells. Troponin T-positive myotubes were decreased by *Wnt3a *overexpression, but not *Wnt4*. TOP/FOP reporter assays revealed that co-expression with *Wnt4 *reduced *Wnt3a*-induced luciferase activity, suggesting that Wnt4 signaling counteracted Wnt3a signaling in myoblasts. FH535, a small-molecule inhibitor of β-catenin/Tcf complex formation, reduced basal β-catenin in the cytoplasm and decreased myoblast proliferation. K252a, a protein kinase inhibitor, increased both cytosolic and membrane-bound β-catenin and enhanced myoblast fusion. Treatments with K252a or Wnt4 resulted in increased cytoplasmic vesicles containing phosphorylated β-catenin (Tyr654) during myogenic differentiation.

**Conclusions:**

These results suggest that various Wnt ligands control subcellular β-catenin localization, which regulate myoblast proliferation and myotube formation. Wnt signaling via β-catenin likely acts as a molecular switch that regulates the transition from cell proliferation to myogenic differentiation.

## Background

Wnt signaling plays key roles in stem cell maintenance and adult tissue homeostasis [1,2]. In addition, Wnt signaling controls cell proliferation and differentiation, as well as organized cell movements and tissue polarity establishment. Wnt signaling dysregulation can induce degenerative and cancerous disorders. The Wnt signaling pathway has gained attention as a potential therapeutic target for cancer treatment, as well as research interest in regenerative medicine and stem cell biology.

Members of the Wnt family are involved in various stages of skeletal muscle development and regeneration [3]. *Wnt1 *and *Wnt3a *expression in the developing neural tube initiate myogenic differentiation in dorsal and medial somites [4,5]. *Wnt3a *overexpression significantly decreases terminally differentiated myogenic cells and causes chick limb malformation by inhibiting chondrogenesis [6,7]. In chick embryos, *Wnt4 *is expressed in developing limbs, particularly in the central elbow region and joint interzones of the wrist-forming region [8]. *Wnt4 *overexpression induces muscle satellite cell markers *Pax7 *and *MyoD*, and increases skeletal muscle mass in chick embryos [9]. *Wnt5a *and *Wnt11 *have been implicated in varying the number of fast and/or slow myofiber types; *Wnt5a *increases and decreases, the number of slow and fast myofibers, respectively, whereas *Wnt11 *has a reversion activity on myofiber specification [6]. Compared to the characterization of these Wnt ligands, intracellular Wnt signaling cooperation during skeletal muscle development and homeostasis is not fully understood.

Wnt family proteins consist of two subfamilies based on downstream intracellular signaling. The canonical Wnt pathway stabilizes β-catenin and activates target genes via TCF/Lef transcription factors. Other Wnt pathways are independent of β-catenin signaling and known as non-canonical Wnt pathways that include stimulation of intracellular Ca2^+ ^release and activation of phospholipase C and protein kinase C. Non-canonical signaling pathways also activate G proteins, RhoGTPases and c-Jun N-terminal kinase (JNK). A recent studies showed that the β-catenin pathway is inhibited by Ror that contains extracellular immunoglobulin (Ig)-like, frizzled-like cysteine-rich, kringle, cytoplasmic tyrosine kinase and proline-rich domains [10]. Ror2 negatively regulates the β-catenin pathway at the TCF-mediated transcription level and activates JNK [11]. The Wnt/Ror pathway is considered to be involved in non-canonical pathways.

Previously, we demonstrated that *Wnt4 *overexpression increases skeletal muscle mass in chick embryos [9]. Wnt4 signaling pathway involvement in skeletal muscle development has been debated, although the level of involvement is dependent on the cell type and context of other regulatory influences. Indeed, Wnt4 can function via the canonical Wnt/β-catenin signaling pathway [12], whereas Wnt4 is mediated by JNK in frog eye and human kidney development [13-15]. While Wnt4 functions are well defined, the underlying mechanisms that regulate expression remain largely unknown.

In this study, we investigate Wnt signaling during differentiation of C2C12 cells that can differentiate into contractile myotubes under a low-serum condition [16]. We use microarray analysis to identify the expression profile of Wnt signaling components during myoblast differentiation. In addition, several Wnt members were overexpressed in C2C12 cells to assess their roles in myoblast differentiation. We also examined small-molecule inhibitor effects on Wnt signaling to evaluate β-catenin/TCF complex and membrane-bound β-catenin involvement in regulating cell proliferation and myoblast differentiation.

## Results

### Expression of Wnt signaling molecules during myogenic differentiation of C2C12 cells

Mouse mesenchymal C2C12 cells can differentiate into muscle cells under a low-serum condition [16]. Using real-time PCR, we investigated the expression of endogenous Wnt signaling components before and after C2C12 cell differentiation. Expression profiles of cells cultured in proliferation medium were used as controls for comparisons to the second and fourth days of culture in differentiation medium.

**Figure 1:**
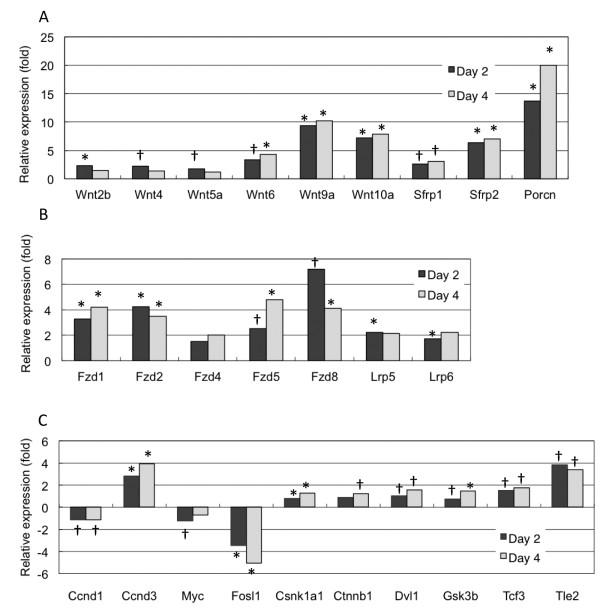
**Endogenous Wnt signaling component expression during myogenic differentiation of C2C12 cells**. Wnt signaling molecule expression was measured by quantitative RT-PCR. After 2 or 4 days culture in differentiation medium, cells were harvested, total RNA prepared and relative gene expression quantified by real-time PCR as follows: (A) wingless-related MMTV integration, *Wnt*; secreted frizzled-related protein, *Sfrp*; and porcupine, *Porcn*. (B) frizzled, *Fzd*; and low density lipoprotein receptor-related protein, *Lrp*. (C) cyclin D, *Ccnd*; myelocytomatosis oncogene, *Myc*; fos-like antigen 1, *Fosl1*; casein kinase 1, alpha 1, *Csnk1a1*; catenin, beta 1, *Ctnnb1*; dishevelled 1, *Dvl1*; glycogen synthase kinase 3 beta, *Gsk3b*; transcription factor 3, *Tcf3*; and transducin-like enhancer of split 2, *Tle2*. Day 2 and 4 time points were normalized to expression levels at day 0. Data are from three independent experiments, *P < 0.005 and † P < 0.05.

*Wnt9a, Wnt10a *and *Wnt6 *expression increased up to day 4, while *Wnt2b*, *Wnt4 *and *Wnt5a *expression reached a maximum at day 2 (Figure 1). Net expression levels of Wnt members were as follows in descending order: Wnt10a > Wnt9a > Wnt2b > Wnt4, Wnt6 > Wnt5a. This expression pattern was consistent with the expression of downstream signaling molecules involved in non-canonical Wnt signaling. Among transmembrane receptors, *frizzled2 *and *frizzled8 *were expressed during early differentiation stages compared with those of *frizzled1*, *frizzled4 *and *frizzled*5. For canonical Wnt signaling target genes, *fosl1 *and *c-myc *expression were decreased, while *Tle2*, a negative regulator of canonical signaling, was increased compared with those of *β-catenin (Ctnnb1), TCF7 *and *Apc*. *Cyclin D1 *and c-*myc *expression decreased, while *cyclin D3 *and *Gsk3β *increased during differentiation [17]. This was consistent with a report that describes Gsk3β regulation of cyclin D1 degradation and subcellular localization [18]. We also found that *porcupine (Porcn) *expression was 13-fold and 19-fold higher at days 2 and 4, respectively, compared with those of cells in a proliferative state (Additional files 1, 2, 3). *Porcn *encodes an *O-*acyltransferase involved in lipid modification of Wnt proteins in the endoplasmic reticulum. Thus, *Porcn *upregulation indicated that Wnt protein transport and secretion was stimulated during myoblast differentiation. Wnt signaling downstream also dynamically changed toward non-canonical pathways following myogenic differentiation.

### Wnt-4 enhances myogenic differentiation of C2C12 cells and antagonizes the canonical Wnt signaling pathway

Next, we evaluated myogenic differentiation induced by Wnt family members. Expression plasmids containing *Wnt3a*, *Wnt4*, *Wnt5a*, *Wnt6*, *Wnt7a*, *Wnt9a *and *Wnt10a *cDNAs were transfected into C2C12 cells. Differentiation was determined by troponin T immunostaining. Although transfection efficiency as indicated by HA-tag immunostaining was ~10%, troponin T expression in *Wnt4*-transfected cells was 3.5-fold higher compared with that of the control (Additional file 4). Troponin T expression was also significantly increased with *Wnt6*, *Wnt7a *and *Wnt9a *overexpression, but not *Wnt3a*, *Wnt5a *and *Wnt10a*. Similar results were also obtained with immunostaining, when skeletal muscle myosin heavy chain was used as a marker protein in place of troponin T (data not shown).

**Figure 2:**
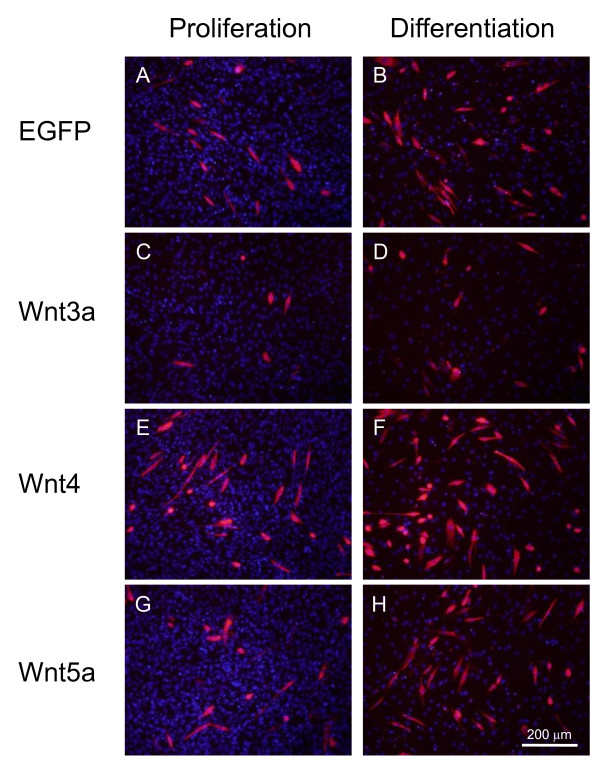
**Wnt4 promotes myogenic differentiation of C2C12 cells**. Expression constructs of Wnt family members in adenovirus vector were transfected into C2C12 cells and myogenic differentiation was determined by troponin T immunostaining after 2 days culture in differentiation medium, which commenced 24 h post-transfection.

**Figure 3:**
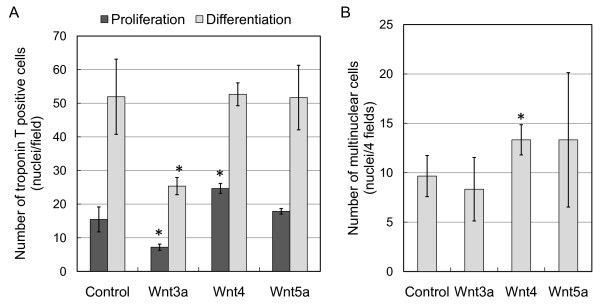
**Wnt4 promotes myogenic differentiation and induces fusion of C2C12 cells**. (A) Troponin T expression after 3 day culture in proliferation or differentiation medium with recombinant adenoviruses expressing *Wnt3a*, *Wnt4*, *Wnt5a *or *eGFP *(control). Cell numbers are the means and standard deviations of three independent experiments performed in triplicate, *P < 0.01 vs. control. (B) Differentiated myotubes were evaluated by counting nuclei within fused multinucleated cells expressing troponin T. Four fields in triplicate cultures were used for counting fused cells cultured under a differentiation condition, *P < 0.04 vs. control.

DNA transfection by cationic liposomes is generally less effective compared with that of recombinant virus infection concerning proportion of the cells expressing HA-tagged Wnt proteins. Therefore, we evaluated myogenic activity using a recombinant adenovirus encoding *Wnt3a*, *Wnt4 *and *Wnt5a*. Within the *Wnt *family, we selected *Wnt3a*, *Wnt4 *and *Wnt5a*, because these *Wnts *exhibit significantly varying biological activities in chick embryos [6-9], although endogenous expression levels of *Wnt3a *and *Wnt5a *in C2C12 cells was less than one-tenth of *Wnt4 *(Additional files 2, 3). C2C12 cells cultured in 24 well plates were infected by the recombinant adenovirus with a multiplicity of infection of 400, because C2C12 cells lack coxsackie and adenovirus receptor expression. Under these conditions, uniform weak expression of the transgene was usually detected at 48 h post-infection. Troponin T immunostaining showed that *Wnt4*-overexpressing cells expressed troponin T and differentiated into myofibers (Figure 2). However, cells that overexpressed *Wnt3a *showed decreased troponin T staining and poor differentiation compared with that of the control expressing *eGFP*. *Wnt5a *overexpression did not significantly affect myoblast differentiation and myofiber formation. *Wnt3a *overexpression significantly reduced troponin T-positive cells in proliferation and differentiation medium (Figure 3A). However, *Wnt4 *overexpression significantly increased troponin T-positive cells in proliferation medium. In differentiation medium, *Wnt4 *overexpression enhanced myotube formation as indicated by fused multinuclear troponin T-positive cells (Figure 3B). *Wnt5a *overexpression demonstrated no effect on C2C12 differentiation under identical conditions.

**Figure 4:**
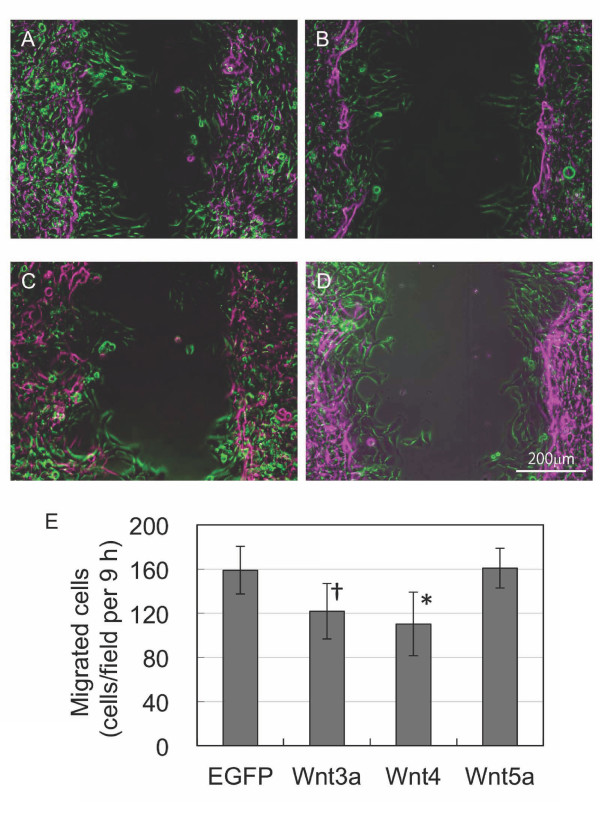
**Effect of Wnt expression on cell migration**. A scratch test was used to determine C2C12 cell proliferation and migration. Cell numbers were counted over 9 h, and mobility was monitored by time-lapse microscopy. Phase contrast images at 0 h (purple) and 9 h (green) were merged to show the migration of C2C12 cells expressing *eGFP *(A), *Wnt3a *(B), *Wnt4*(C) and *Wnt5a *(D). (E) Summary of cell numbers counted over 9 h (cells/field) with n = 3 for control, Wnt3a and Wnt4, and n = 4 for Wnt5a. *P < 0.01; † P < 0.02 vs. eGFP.

A scratch wound-healing assay was performed to examine proliferation and migration of cells overexpressing *Wnt3a*, *Wnt4 *and *Wnt5a*. After 48 h post-transfection, C2C12 cell monolayers were scratched to remove cells along the middle of the culture plate surface. Then, cell migration was monitored by time-lapse video microscopy (Additional files 5, 6, 7, 8). Cellular migration of *Wnt5a*-expressing cells was nearly identical to that of control cells expressing enhanced green florescent protein *(eGFP)*, while *Wnt4*- and *Wnt3a*-expressing cells showed decreased migration to 69% and 77%, respectively (Figure 4). Although we observed cell migration in the scratch assay, we could not detect any differences between *eGFP *and *Wnt5a *transgenes during regeneration of the cells at the wound edges.

**Figure 5:**
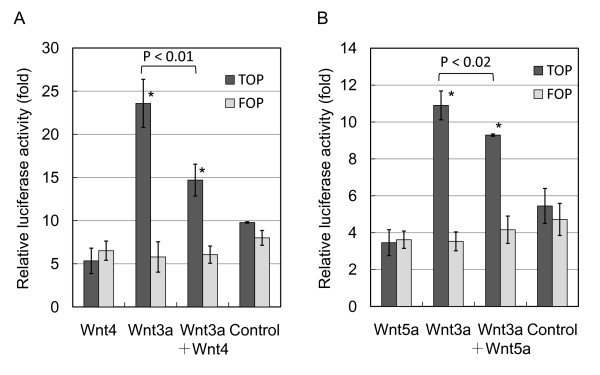
**Wnt4 reduces Wnt-3a-induced transcriptional activity in myoblasts**. Reporter assays were performed using TOP/FLASH and FOP/FLASH (luciferase assay) in C2C12 cells. *Wnt3a *significantly increases luciferase activity in TOP over FOP. (A) *Wnt4 *has no effect on luciferase activity, whereas *Wnt4 *inhibits *Wnt3a *activity. (B) *Wnt5a *poorly inhibits *Wnt3a *activity. Bars are the means of four independent experiments. *P < 0.01 between TOP and FOP; P < 0.01 between *Wnt3a *and *Wnt3a *+ *Wnt4 *in TOP; P < 0.02 between *Wnt3a *and *Wnt3a *+ *Wnt5a *in TOP.

We then examined whether Wnt4 mediated myogenesis induction by canonical or non-canonical signaling using a TOP/FOP reporter assay in C2C12 cells under a proliferative condition. *Wnt4 *and *Wnt3a *reporter constructs were transfected into C2C12 cells and luciferase activity was determined at 24 h post-transfection. Although *Wnt3a *significantly elevated reporter activity, *Wnt4 *did not show increased activity above FOP (Figure 5A). However, *Wnt4 *coexpressed with *Wnt3a *showed 47% inhibition. Weak inhibition was observed when *Wnt5a *was coexpressed with *Wnt3a *(Figure 5B). These results indicated that Wnt4 suppressed canonical Wnt signaling mediated by the β-catenin/TCF complex and promoted myogenic differentiation. This observation was consistent with down-regulated *cyclin D1 *and *c-myc *in differentiating myoblasts, both of which are direct targets of the β-catenin/TCF complex.

### β-Catenin is a molecular switch between C2C12 cell proliferation or differentiation

**Figure 6:**
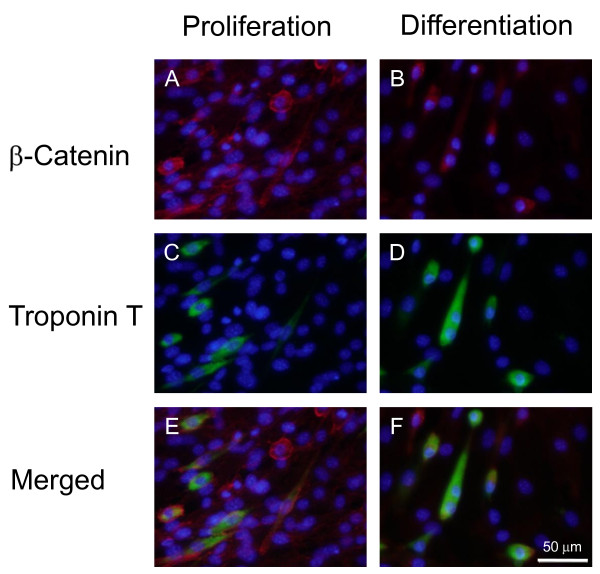
**Localization of β-catenin and troponin T in proliferative and differentiating C2C12 cells**. C2C12 cells cultured in proliferation and differentiation medium were double immunostained for β-catenin (A, B; red) and troponin T (C, D; green), and counterstained with DAPI (blue). Immunofluorescent images with various filters are merged (E, F).

Wnt4, represented by luciferase activity, inhibited transcriptional activation via β-catenin/TCF. In HEK-293T cells, Wnt4 relocates β-catenin to the cell membrane [19]. We observed subcellular β-catenin localization by immunohistochemistry in C2C12 cells cultured in proliferation and differentiation medium (Figure 6). Under a proliferative condition, β-catenin was predominantly localized in the cytoplasm. β-Catenin was also localized within the nucleus of round proliferating cells and weak β-catenin was coexpressed with differentiation marker troponin T (Figure 6E). This observation coincided with changing morphology to flattened multinucleated cells. In differentiation medium, cellular β-catenin accumulated in the membrane and nuclei as differentiation proceeded into myotubes (Figure 6B,F).

**Figure 7:**
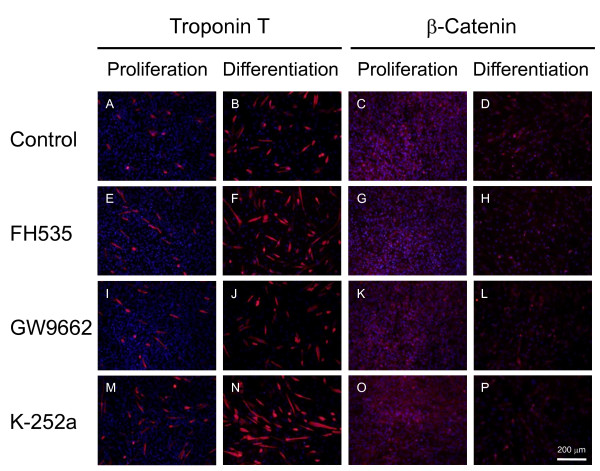
**Effect of small-molecule inhibitors on C2C12 cell proliferation and differentiation**. C2C12 cells were cultured without inhibitors (A-D) and with 1 μM FH535 (E-H), 1 μM GW9662 (I-L) and 3.6 nM K-252a (M-P), then immunostained with anti-troponin T (A, B, E, F, I, J, M, N) and anti-β-catenin (C, D, G, H, K, L, O, P) antibodies. FH535 interferes with β-catenin/Tcf complex formation, whereas GW9662 is structurally similar to FH535, but does not interfere with complex formation. K252a is a Trk kinase inhibitor, which phosphorylates β-catenin at tyrosine 654.

**Figure 8:**
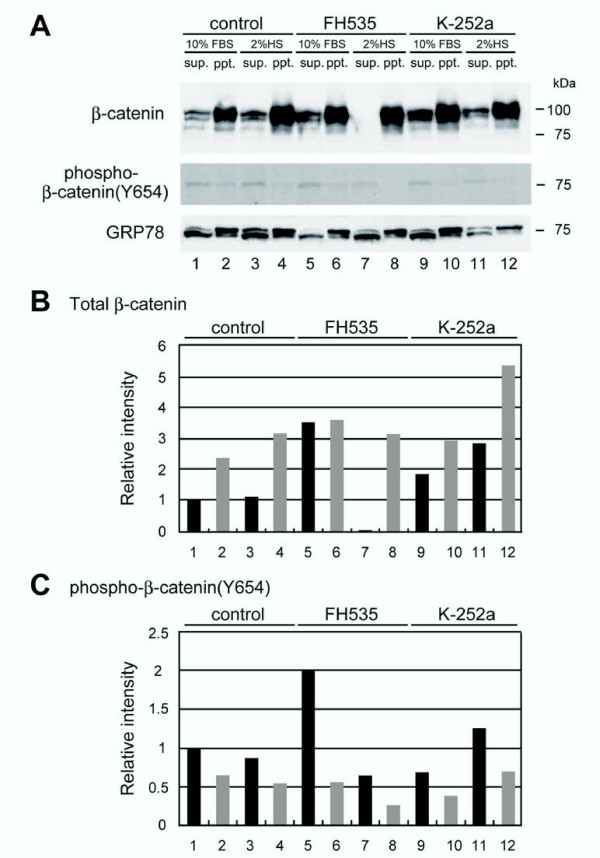
**FH535 and K252a alter total and phospho-β-catenin (Y654) in C2C12 cells**. (A) Western blots of cytosolic and membrane fractions from C2C12 cells treated with FH535 and K-252a using antibodies against total cellular-β-catenin (upper panel) and phosphorylated β-catenin (lower panel). GRP78 indicates loading control. (B, C) Relative levels of total and phosphorylated β-catenin in cytosolic (black) and membrane (grey) fractions were calculated as the β-catenin/GRP78 ratio. Band intensities are relative to the control cell values in the cytosolic fraction and defined as 1.0. Similar results were obtained in three independent experiments.

**Figure 9:**
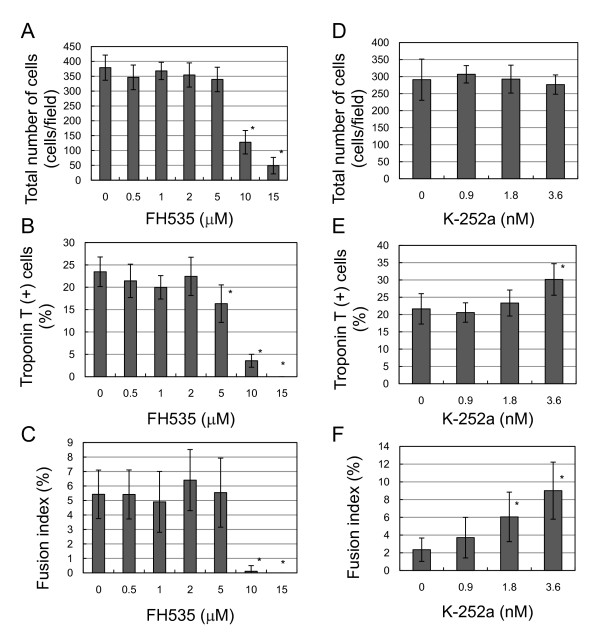
**Effect of FH535 and K252a on C2C12 cell proliferation and differentiation**. C2C12 cells were cultured with or without various FH535 and K252a concentrations followed by troponin T immunostaining. Total cell numbers are the counted DAPI-stained nuclei and the fusion index is the percentage of multinucleated cells among troponin T-positive cells. *P < 0.001 vs. absence of inhibitors.

Small-molecule inhibitors of Wnt signaling have been developed for human cancer therapy and allow manipulation of intracellular signaling to decrease cell proliferation and induce differentiation or apoptosis [20]. To investigate Wnt signaling and β-catenin during C2C12 myoblast differentiation, we used chemical inhibitors FH535, GW9662 and K252a to observe troponin T expression and subcellular β-catenin localization by immunohistochemistry (Figure 7) and western blotting (Figure 8). FH535 interferes with β-catenin/Tcf complex formation, whereas GW9662 is structurally similar to FH535 but does not interfere with complex formation. K252a is a Trk kinase inhibitor, which phosphorylates β-catenin at tyrosine 654 [21,22]. FH535 and GW9662 did not affect myoblast differentiation at 1 μM, although FH535 exhibited cytotoxicity at concentrations > 10 μM (Figure 9). K252a dose-dependently increased troponin T-positive cells and promoted myoblast fusion, as reported elsewhere [21]. Subcellular β-catenin localization was determined by western blot analysis of cytoplasmic and membrane extracts from proliferative and differentiating C2C12 cells (Figure 9). FH535 increased the cytosolic level of β-catenin in proliferation medium, but decreased basal β-catenin in differentiation medium (Figure 8A, B). In contrast, K252a increased cytosolic and membrane-bound β-catenin during myogenic differentiation (Figure 9B).

**Figure 10:**
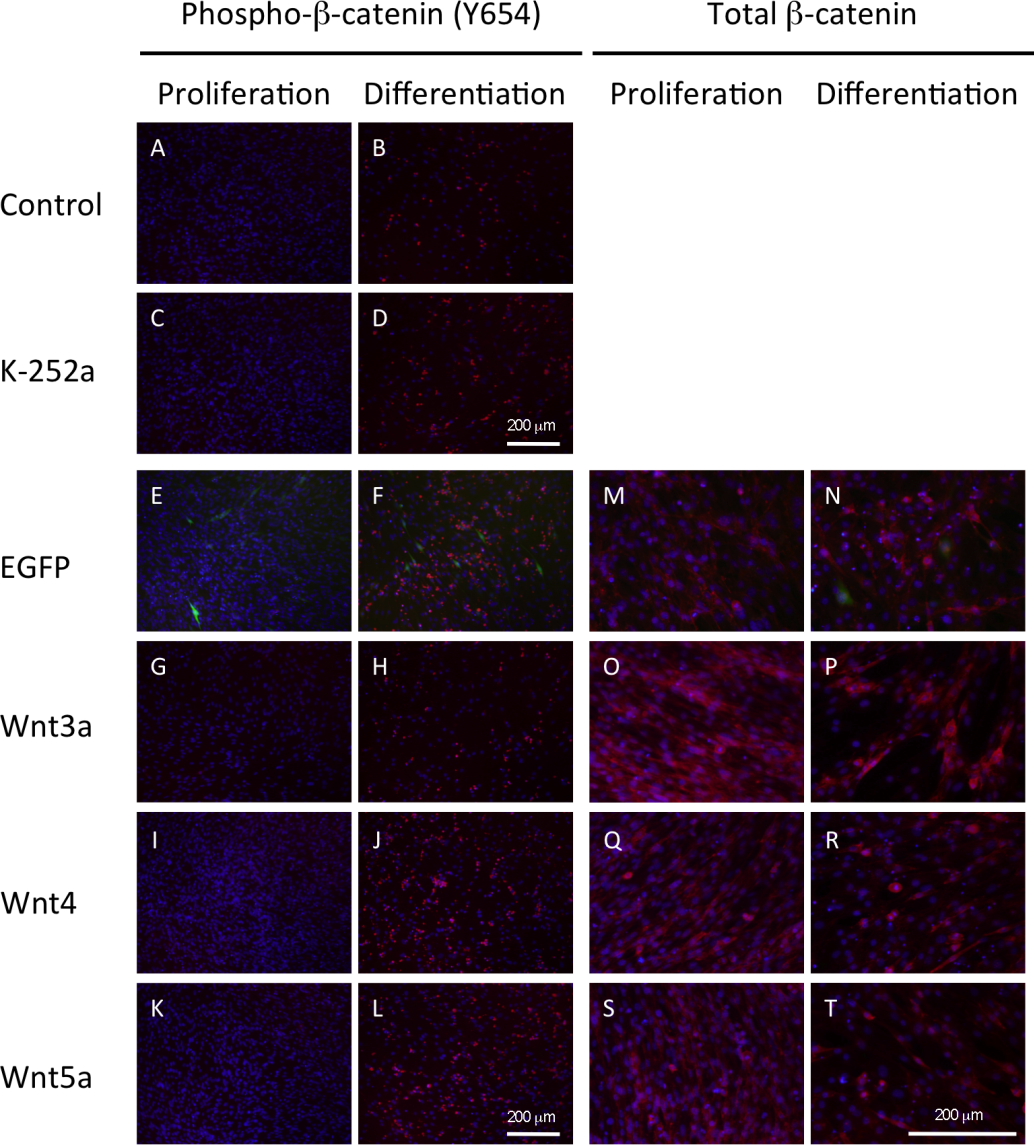
**Wnt family members differentially regulate β-catenin phosphorylation and subcellular localization in C2C12 cells**. (A-D) C2C12 cells were cultured with or without K252a, and then phospho-β-catenin (Y654) was immunostained. K252a caused an accumulation of vesicles containing phospho-β-catenin (Y654) in C2C12 cells. (E-L) *Wnt3a*, *Wnt4 *and *Wnt5a *are overexpressed in C2C12 cells by adenovirus-mediated gene transfer. After 2 day culture in differentiation medium commencing 24 h post-transfection, subcellular phospho-β-catenin (Y654) localization was determined by immunostaining. (M-T) C2C12 cells expressing *eGFP*, *Wnt3a*, *Wnt4 *and *Wnt5a *were immunostained with anti-pan-β-catenin antibodies. Cytoplasmic β-catenin is elevated with *Wnt3a *expression in proliferation medium and maintained at a higher level in differentiation medium, whereas nuclear and membrane-bound β-catenin is increased with *Wnt4 *expression.

Next, we analyzed β-catenin phosphorylation at residue Y654 during myoblast proliferation and differentiation. Proliferation medium supplemented with FH535 increased phospho-β-catenin (Y654) by 2-fold, but decreased phospho-β-catenin to less than half in differentiation medium (Figure 8C). In contrast, K252a-supplemented proliferation medium decreased phospho-β-catenin (Y654) as anticipated, but increased phosphorylated β-catenin and total β-catenin in differentiation medium. This observation was consistent with the immunohistochemical analysis. K252a treatment in differentiation medium caused cytosolic accumulation of small vesicles containing phospho-β-catenin (Y654) (Figure 10A,B). Together with western blot data, these results suggested that K252a increases total β-catenin in C2C12 cells. Simultaneously, β-catenin may be phosphorylated at tyrosine 654 by a kinase that was not inhibited by K252a and induced in differentiation medium via serum starvation.

C2C12 cells overexpressing *Wnt3a*, *Wnt4 *and *Wnt5a *were stained with the same monoclonal antibody to determine cellular phospho-β-catenin in differentiation medium. Consistent with troponin T expression, decreased vesicles containing phospho-β-catenin (Y654) were observed in *Wnt3a*-overexpressing cells cultured in differentiation medium, whereas *Wnt4*- and *Wnt5a*-overexpressing cells contained increased vesicles compared with that of the control cells expressing *eGFP *(Figure 10F, H, J, L).

Lastly, we analyzed localization of β-catenin within cells overexpressing *Wnt3a*, *Wnt4 *and *Wnt5a*. *Wnt3a *overexpression resulted in an elevated cytoplasmic β-catenin level in both proliferation and differentiation mediums (Figure 10O, P). In contrast, *Wnt4 *and *Wnt5a *overexpression showed an increase in nuclear and membrane-bound β-catenin levels (Figure 10Q, R, S, T). Collectively, our results suggested that Wnt family members differentially regulate β-catenin phosphorylation and subcellular localization. Moreover, β-catenin is required to maintain cell proliferation and acts as a molecular switch that regulates myogenic differentiation.

## Discussion

Wnt/β-catenin signaling is essential for skeletal muscle development and regeneration. Wnt signals via β-catenin are necessary to induce muscle-specific gene transcription in P19 embryonic carcinoma cells [23]. Canonical Wnt signaling also promotes muscle satellite-cell proliferation in response to skeletal muscle injury [24]. The β-catenin/TCF complex binds *cyclin D1 *and c-*myc *promoters, and increases expression in carcinoma cells. However, β-catenin/TCF target gene expression is decreased in differentiating C2C12 myoblast cells. C*yclin D1, c-myc *and *fosl1 *down-regulation as well as a compensating *cyclin D3 *elevation suggest pathway switching from β-catenin dominant signaling to other pathways. We demonstrated that Wnt family members, including *Wnt2b*, *Wnt4*, *Wnt6*, *Wnt9a*, and *Wnt10a*, are significantly elevated following myogenic differentiation of C2C12 cells. Within the Wnt family, *Wnt4*, *Wnt6*, *Wnt7a*, and *Wnt9a *induce myogenic differentiation following transient overexpression in C2C12 cells, whereas *Wnt3a*, *Wnt5a*, and *Wnt10a *do not affect troponin T expression. *Wnt4 *overexpression optimally promotes myogenesis and antagonizes transcription mediated by β-catenin/TCF. We therefore selected Wnt3a, Wnt4 and Wnt5a to analyze canonical and/or non-canonical signaling during myogenic differentiation of C2C12 cells.

We found that Wnt4 suppresses canonical Wnt signaling mediated by the β-catenin/TCF complex and promotes myogenic differentiation. However, numerous reports indicate that activation of the Wnt/β-catenin signaling pathway increases myogenic differentiation directly or indirectly [24-30]. One possible explanation may be that canonical Wnt signaling maintains proliferation of myoblast and progenitors, such as satellite cells and reserve cells, and subsequently induces myogenic differentiation. Indeed, canonical Wnt signaling downstream targets *cyclin D1 *and *c-myc *are suppressed during myoblast differentiation [17] (Figure 1). This observation is consistent with *c-myc *overexpression in C2C12 cells resulting in inhibition of myoblast fusion and myotube formation [31]. Moreover, increased canonical Wnt signaling is related to skeletal muscle aging [32,33]. Further analyses of the cell cycle and β-catenin are required to characterize canonical Wnt signaling during myogenic differentiation.

Recent studies have shown that Wnt4 activates the canonical β-catenin pathway in C2C12 cells [30]. This difference of results may be partly due to cell culture conditions and C2C12 cell characteristics. First, the C2C12 cell cycle is not synchronized with medium replacement and various cell cycle phases are observed, as shown in Figure 6. Second, serum components in the medium may affect the results, because proliferation and differentiation of C2C12 cells is controlled by lysophosphatidic acid and cholesterol [34,35]. Third, C2C12 cell characteristics may substantially change during serial passaging. Therefore, Wnt4 could mediate either canonical or non-canonical signaling pathways and overexpression may promote myogenic differentiation of C2C12 cells. During the transition from cell proliferation to differentiation in C2C12 cells, β-catenin expression is down-regulated and troponin T expression is predominantly observed in β-catenin-negative cells (Figure 6). Therefore, β-catenin signaling may be required during early myogenic differentiation stages and unnecessary or inhibitory toward myotube formation after myogenic determination.

Functional switching from cell proliferation to myogenic differentiation is accompanied by changes in cell migration, as revealed by a scratch test analysis (Figure 4). Maintenance of an undifferentiated state with Wnt3a overexpression results in decreased migration, while transition toward myogenic differentiation further reduces migration possibly via non-canonical pathways. Wnt3a activity via the canonical pathway affects cell survival and proliferation. Additionally, Wnt3a is required for maintenance of the myoblast undifferentiated state as well as differentiation induced by Wnt4 signaling.

Our results suggest that β-catenin acts as a molecular switch between proliferation and differentiation of C2C12 cells. The β-catenin/Tcf complex is essential for C2C12 cell proliferation, and inhibition of complex formation by FH535 promotes cell death, although the detailed mechanism of FH535 inhibition is unknown at present. β-Catenin is phosphorylated by various kinases, which causes β-catenin degradation or signaling activity. K252a inhibition of Y654 phosphorylation shifts β-catenin localization from the cytoplasm to the cell membrane, which stimulates myotube formation (Figure 10). Wnt4 induces β-catenin shuttling from the nucleus to the cytoplasm and the regulatory mechanism is not known.

## Conclusions

We found that various Wnt ligands regulate differentially subcellular β-catenin localization as well as myoblast proliferation and myotube formation. These results implicate β-catenin in functional switching from cell proliferation to myogenic differentiation.

## Methods

### Plasmid construction, cell culture and transfection assay

*eGFP *cDNA was purchased from Wako Chemicals as GFP pQBI-polII. *Wnt4 *and *eGFP *cDNAs were subcloned into pcDNA3.2DEST (Invitrogen, Carlsbad, CA) for overexpression in C2C12 cells. A HA-tag was added to the *Wnt4 *C-terminal using mouse *Wnt3a *cDNA in pUSEamp (Upstate Biotechnology Inc., Temecula, CA) that contained the HA-tag sequence at the C-terminal end, after subcloning to replace the *Wnt3a *sequence with *Wnt4*. All *Wnt *cDNAs used in the present studies contained HA-tag sequence at the C-terminal end. *Wnt3aHA*, *Wnt4HA*, *Wnt5aHA*, *Wnt6HA*, *Wnt7aHA*, *Wnt9aHA*, *Wnt10aHA *and *eGFP *(Wako Chemicals, Osaka, Japan) cDNAs were subcloned into pcDNA3.2 (Invitrogen) for transfection and transient expression.

The C2C12 cell line (myoblast-like cell line from the C3H mouse) was purchased from the RIKEN Cell Bank (RIKEN, Wako, Japan) [16,36] and cultured in Dulbecco's modified Eagle's medium (D-MEM) supplemented with 10% fetal bovine serum (FBS). At 12-24 h after subculture, transfection was performed using Lipofectamine 2000 according to the manufacturer's instructions (Invitrogen). Transfected C2C12 cells were cultured in differentiation medium consisting of 2% horse serum in D-MEM to induce myogenesis.

### Real-time quantitative PCR

C2C12 cells were harvested at days 2 (n = 3) and 4 (n = 3) after replacement of proliferation medium with differentiation medium. Total RNA was extracted from C2C12 cells using ISOGEN (NIPPON GENE CO., Tokyo, Japan) according to the manufacturer's instructions. RNA was reverse transcribed into cDNA (RT^2 ^First Strand Kit, SABiosciences) and the cDNA used for quantitative PCR analysis. Wnt signaling component expression in C2C12 cells was analyzed by quantitative Real-Time RT-PCR using a SA Biosciences RT^2^Profiler™ PCR Array (http://www.sabiosciences.com/rt_pcr_product/HTML/PAMM-043A.html) and RT^2 ^SYBR Green Master Mixes. The PCR condition was 95°C for 10 min followed by 40 cycles of 95°C for 15 sec and 60°C for 1 min. Analyses were performed on an ABI Real-Time PCR System 7500 (Applied Biosystems, Foster City, CA). Data were evaluated by the ΔΔCt method against control cells cultured in proliferation medium (n = 4), using an on-line template (http://pcrdataanalysis.sabiosciences.com/pcr/arrayanalysis.php). ΔCt values were calculated by subtracting average cycle threshold (Ct) values of housekeeping genes (glucuronidase, beta, Gusb; Heat shock protein 90 alpha (cytosolic), class B member 1, Hsp90ab1; glyceraldehyde-3-phosphate dehydrogenase, Gapdh; actin, beta, Actb; and hypoxanthine guanine phosphoribosyl transferase 1, Hprt1). MIAME (Minimum Information About a Microarray Experiment) compliant array data including raw data is deposited in GEO (Gene Expression Omnibus) at NCBI [GSE30077].

### Viral vector production and treatment

Adenoviruses carrying *Wnt *cDNAs were prepared using a ViraPower adenovirus expression system (Invitrogen). Human *Wnt3a*, *Wnt4 *and *Wnt5a *cDNAs (Origene, Rockville, MD) were PCR-amplified and subcloned into a pAd/CMV/V5-DEST vector (Invitrogen) using a Gateway system with LR clonase (Invitrogen). *eGFP *cDNA was used as a control after subcloning into a pAd/CMV/V5-DEST vector. Plasmids were purified and digested with *Pac*I (New England Biolabs Japan Inc., Tokyo, Japan). Linearized plasmids (1-2 μg) were then mixed with 3 μl Lipofectamine 2000 in 200 μl Opti-MEM medium (Invitrogen) and transfected into subconfluent 293A cells (Invitrogen) cultured in 1 ml Opti-MEM in 35 mm plates (Nunc, Thermo Fisher Scientific, Yokohama, Japan). 293A cells were cultured in 100 mm plates for 2 weeks in proliferation medium that was exchanged every 3 days. Cells and medium were harvested upon cell detachment from culture plates, freeze-thawed four times and then centrifuged to obtain adenovirus-enriched supernatants. Aliquots of supernatant were added to fresh 293A cells and cultured for 2-3 days to propagate the adenovirus. After 2-fold amplification, adenovirus-containing medium was used as the virus stock. Viral titers were determined by a plaque-forming assay using 293A cells.

### Reporter assays

C2C12 cells were seeded into 24 well plates at 2.5 × 10^4 ^cells/well and cultured for 24 h. Cells were then cotransfected with 0.2 μg DNA constructs (Wnt3aHA + pcDNA3.2, Wnt4HA + pcDNA3.2, Wnt3aHA + Wnt4HA), 0.2 μg reporter plasmid (TOPFLASH, FOPFLASH) and 0.2 μg internal control pRG-TK, then cultured for a further 24 h. Luciferase activity was measured and normalized for transfection efficiency using a Dual-Glo luciferase assay system (Promega, Madison, WI). Graphs show the average of three independent experiments with normalized transfection efficiency using Rluc.

### Scratch test analysis

Scratch testing was performed using C2C12 cells transfected with *Wnt3a*, *Wnt4*, *Wnt5a *and *eGFP *in pcDNA3.2, which were cultured in 35 mm dishes. Two days after transfection, the cell monolayer was scratched using a toothpick. Cell proliferation and migration were recorded at 5 min intervals and observed for 16 h using an AQUACOSMOS image acquisition and analysis system (Hamamatsu, Japan). Initial cells counts were subtracted from the 9 h counting.

### Immunofluorescence assays

Cells were fixed in a solution of ethanol: formalin: acetic acid: H_2_O (14:2:1:6, v/v) for 10 min. After three PBS washes, cells were treated with a blocking buffer consisting of 2% goat serum, 2% skim milk, 0.2% Tween20 in Tris-buffered saline (TBS; 50 mM Tris-HCl, pH 7.5, 150 mM NaCl) for 30 min. Then, cells were incubated with anti-troponin T (MAB1487, clone TT-98, Abnova, CA), anti-β-catenin (C2206, Sigma-Aldrich) and anti-phospho-β-catenin (Y654) (ab24925, Abcam, Tokyo, Japan) antibodies at 1:40, 1:200 and 1:50 dilutions, respectively, in blocking buffer at 4°C overnight. After three TBS washes, cells were incubated with a 1:200 dilution of secondary Alexa Fluor 594 or 488-conjugated goat anti-mouse or rabbit IgG antibodies (A11032, A11037, A11029, Molecular Probes, Invitrogen) for 1 h. After a TBS wash, cell nuclei were stained with 1 μg/ml 4', 6'-diamino-2-phenylindole solution (DAPI, DOJINDO, Kumamoto, Japan). Fluorescent images were taken using All-in-One Fluorescence Microscope BZ-9000 (Keyence Japan, Osaka, Japan).

### Inhibitor assays

C2C12 cells were seeded into 24 well plates at 2.5 × 10^4 ^cells/well and cultured for 24 h. Cells were incubated in proliferation or differentiation medium with or without chemical inhibitors FH535 (1 μM), GW9662 (1 μM) and K-252a (3.6 nM).

### Western blot analysis

At 24 h post-subculture, C2C12 cell culture medium was changed to differentiation medium supplemented with FH535 (1 μM) or K-252a (3.6 nM). After 72 h, cells were harvested to prepare total protein extracts.

Cytosolic β-catenin accumulation was assayed as described elsewhere [37,38]. Protein extracts were resolved by electrophoresis in 5-20% sodium dodecyl sulfate-polyacrylamide gels. Anti-β-catenin (BD Transduction Laboratory, San Diego, CA), anti-phospho-β-catenin (Y654) (ab24925, Abcam), and anti-GPR78 (Santa Cruz) antibodies were used at 1:2000, 1:500, and 1:5000 dilutions, respectively. Proteins were detected using an ECL Plus System (Amersham Pharmacia Biotech, Uppsala, Sweden) with a 1:10 reagent dilution. Band intensities were determined using Image J software (NIH USA).

## Competing interests

The authors declare that they have no competing interests.

## Authors' contributions

TN conceived and designed the experiments; ST and KT performed the experiments and analyzed the data; ST and TN prepared the manuscript. All authors read and approved the final manuscript.

## Supplementary Material

Additional file 1 **Real-time PCR analysis**. (A) Array layout of 96 well RT-PCR kit. (B) Heat map of PCR results between day 2 and 0 samples in differentiation medium. (C) Heat map of PCR results between day 4 and 0 samples in differentiation medium. Note: the scales are not identical in panels B and C. Click here for file

Additional file 2 **Real-time PCR results (Day 2 vs control)**. Array results of real-time PCR analysis. n = 3 for the Day 2 group and n = 4 for the control group. Click here for file

Additional file 3 **Real-time PCR results (Day 4 vs control)**. Array results of real-time PCR analysis. n = 3 for the Day 4 group and n = 4 for the control group. Click here for file

Additional file 4 **Effect of Wnt expression on cell differentiation**. C2C12 cells were transfected with *Wnt *cDNAs in pcDNA3.2 and cultured in proliferation medium for 2 days. Cells were fixed, troponin T immunostained and positive cell nuclei were counted. *P < 0.03 vs. control. Click here for file

Additional file 5 **Time-lapse movie (control)**. The movie consists of micrographs taken at 5 min intervals for 16 h. Cells were transfected with an *eGFP*-expressing plasmid. Click here for file

Additional file 6 **Time-lapse movie (Wnt4)**. The movie consists of micrographs taken at 5 min intervals for 16 h. Cells were transfected with a *Wnt4*-expressing plasmid. Click here for file

Additional file 7 **Time-lapse movie (Wnt5a)**. The movie consists of micrographs taken at 5 min intervals for 16 h. Cells were transfected with a *Wnt5a*-expressing plasmid. Click here for file

Additional file 8 **Time-lapse movie (Wnt3a)**. The movie consists of micrographs taken at 5 min intervals for 16 h. Cells were transfected with a *Wnt3a*-expressing plasmid. Click here for file
