# Supplementary material for: Canonical Wnt signaling is involved in switching from cell proliferation to myogenic differentiation of mouse myoblast cells
Source: J Mol Signal. 2011 Oct 5;6:12. doi: 10.1186/1750-2187-6-12 (PMC3198762; doi:10.1186/1750-2187-6-12)

A

	1	2	3	4	5	6	7	8	9	10	11	12
A	Aes	Apc	Axin1	Bcl9	Btrc	Ctnnbip1	Ccnd1	Ccnd2	Ccnd3	Csnk1a1	Csnk1d	Csnk2a1
B	Ctbp1	Ctbp2	Ctnnb1	Daam1	Dixdc1	Dkk1	Dvl1	Dvl2	Ep300	Fbxw11	Fbxw2	Fbxw4
C	Fgf4	Fosl1	Foxn1	Frat1	Frzb	Fshb	Fzd1	Fzd2	Fzd3	Fzd4	Fzd5	Fzd6
D	Fzd7	Fzd8	Gsk3b	Jun	Kremen1	Lef1	Lrp5	Lrp6	Myc	Nkd1	Nlk	Pitx2
E	Porcn	Ppp2ca	Ppp2r1a	Ppp2r5d	Pygo1	Rhou	Senp2	Sfrp1	Sfrp2	Sfrp4	Slc9a3r1	Sox17
F	T	Tcf3	Tcf7	Tle1	Tle2	Wif1	Wisp1	Wnt1	Wnt10a	Wnt11	Wnt16	Wnt2
G	Wnt2b	Wnt3	Wnt3a	Wnt4	Wnt5a	Wnt5b	Wnt6	Wnt7a	Wnt7b	Wnt8a	Wnt8b	Wnt9a
H	Gusb	Hprt1	Hsp90 ab1	Gapdh	Actb	MGDC	RTC	RTC	RTC	PPC	PPC	PPC

B

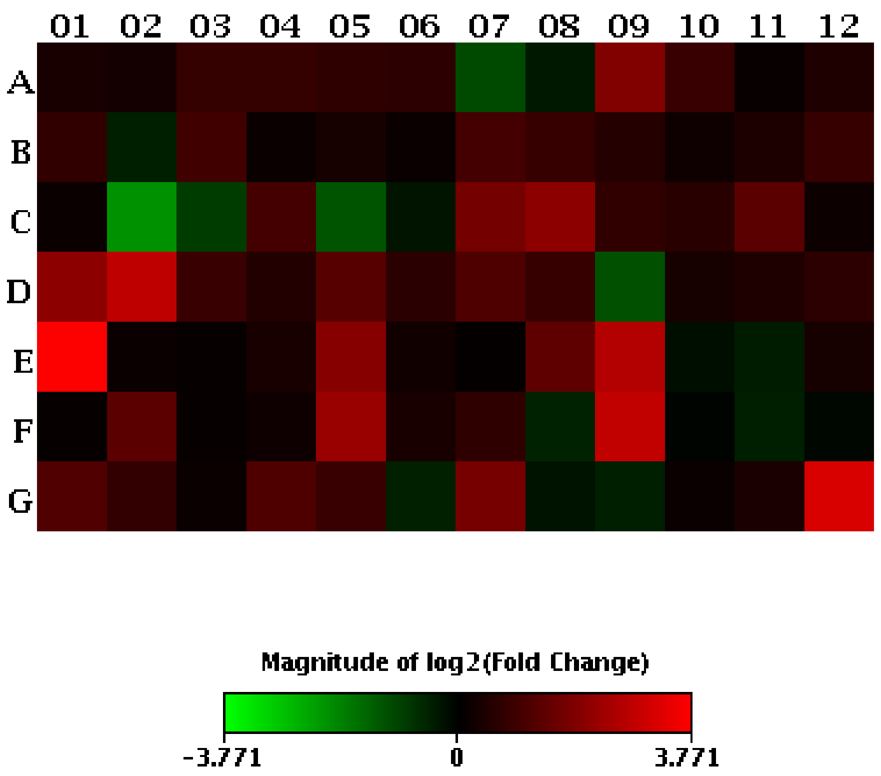

C

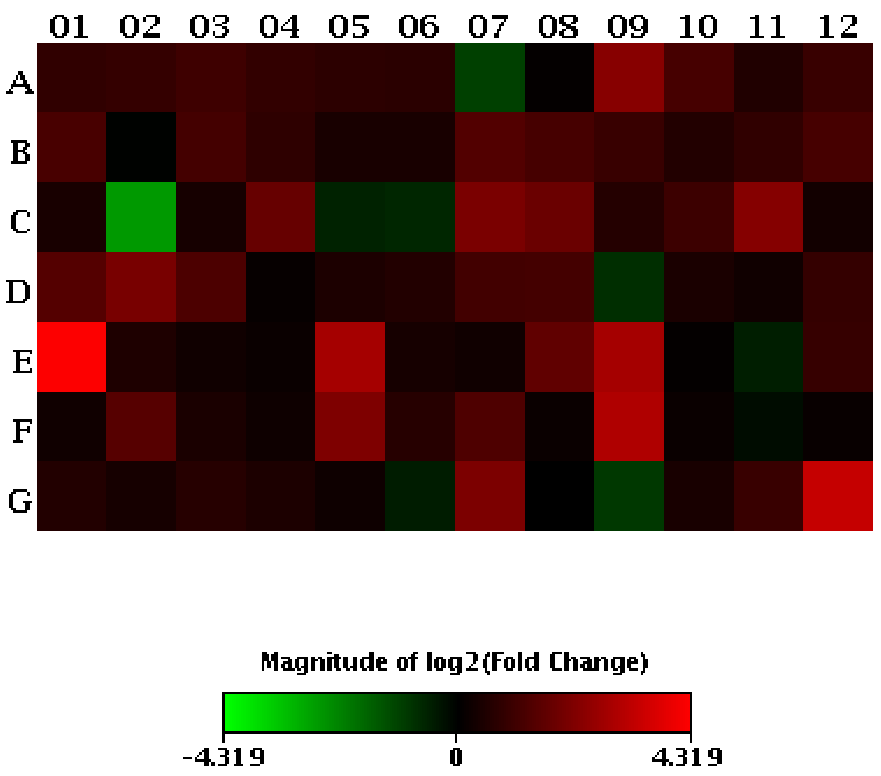

Supplement: Additional file 1 — Real-time PCR analysis. (A) Array layout of 96 well RT-PCR kit. (B) Heat map of PCR results between day 2 and 0 samples in differentiation medium. (C) Heat map of PCR results between day 4 and 0 samples in differentiation medium. Note: the scales are not identical in panels B and C. [file 1750-2187-6-12-S1.PDF]
