# Supplementary material for: Canonical Wnt signaling is involved in switching from cell proliferation to myogenic differentiation of mouse myoblast cells
Source: J Mol Signal. 2011 Oct 5;6:12. doi: 10.1186/1750-2187-6-12 (PMC3198762; doi:10.1186/1750-2187-6-12)
Supplement: Additional file 2 — Real-time PCR results (Day 2 vs control). Array results of real-time PCR analysis. n = 3 for the Day 2 group and n = 4 for the control group. [file 1750-2187-6-12-S2.PDF]

Real time RT-PCR results (Day 2 vs control)

Symbol	$2^{\wedge}\text{-}\Delta C_t$		Fold Difference	T-TEST	Fold Up- or Down-Regulation
	Day 2	Control Sample	Day 2 /Control Sample	p value	Day 2 /Control Sample
Aes	1.3E-01	9.8E-02	1.29	0.3567	1.29
Apc	2.9E-02	2.3E-02	1.24	0.4653	1.24
Axin1	1.5E-02	8.8E-03	1.70	0.2025	1.70
Bcl9	3.5E-03	2.0E-03	1.71	0.0037	1.71
Btrc	6.2E-03	3.9E-03	1.59	0.0057	1.59
Ctnnbip1	1.1E-02	7.0E-03	1.58	0.1978	1.58
Ccnd1	5.7E-02	1.2E-01	0.46	0.0253	-2.15
Ccnd2	7.1E-02	9.1E-02	0.78	0.2394	-1.28
Ccnd3	3.4E-01	8.9E-02	3.82	0.0006	3.82
Csnk1a1	1.9E-01	1.0E-01	1.79	0.0016	1.79
Csnk1d	8.7E-02	8.1E-02	1.08	0.7025	1.08
Csnk2a1	1.7E-01	1.3E-01	1.35	0.0381	1.35
Ctbp1	3.7E-02	2.3E-02	1.63	0.2715	1.63
Ctbp2	5.2E-02	7.1E-02	0.73	0.2747	-1.36
Ctnnb1	2.2E-01	1.1E-01	1.92	0.0523	1.92
Daam1	4.2E-02	3.8E-02	1.12	0.6435	1.12
Dixdc1	1.9E-03	1.5E-03	1.25	0.1197	1.25
Dkk1	6.7E-06	5.9E-06	1.12	0.7580	1.12
Dvl1	1.8E-02	8.8E-03	2.02	0.0303	2.02
Dvl2	1.2E-02	6.9E-03	1.73	0.1588	1.73
Ep300	2.3E-02	1.6E-02	1.43	0.0024	1.43
Fbxw11	3.2E-02	2.7E-02	1.17	0.5975	1.17
Fbxw2	6.6E-02	4.9E-02	1.34	0.1109	1.34
Fbxw4	8.4E-03	4.9E-03	1.73	0.1888	1.73
Fgf4	6.7E-06	5.9E-06	1.12	0.7580	1.12
Fosl1	1.9E-02	8.5E-02	0.23	0.0006	-4.43
Foxn1	3.8E-04	6.9E-04	0.54	0.0150	-1.84
Frat1	7.6E-05	3.8E-05	1.99	0.0535	1.99
Frzb	1.1E-05	2.6E-05	0.42	0.0728	-2.37
Fshb	2.0E-05	2.4E-05	0.81	0.8257	-1.23

Fzd1	4.0E-02	1.2E-02	3.29	0.0007	3.29
Fzd2	9.1E-03	2.1E-03	4.26	0.0039	4.26
Fzd3	1.3E-02	7.9E-03	1.64	0.0194	1.64
Fzd4	1.6E-03	1.0E-03	1.51	0.0571	1.51
Fzd5	1.6E-02	6.2E-03	2.50	0.0065	2.50
Fzd6	9.7E-03	8.6E-03	1.13	0.4128	1.13
Fzd7	4.9E-03	1.1E-03	4.30	0.0671	4.30
Fzd8	1.4E-04	2.0E-05	7.21	0.0057	7.21
Gsk3b	1.0E-02	5.7E-03	1.76	0.0177	1.76
Jun	7.5E-02	5.2E-02	1.42	0.2244	1.42
Kremen1	9.8E-02	4.0E-02	2.44	0.0270	2.44
Lef1	3.9E-03	2.5E-03	1.55	0.1425	1.55
Lrp5	2.4E-02	1.1E-02	2.23	0.0491	2.23
Lrp6	3.4E-02	1.9E-02	1.74	0.0039	1.74
Myc	1.8E-02	4.0E-02	0.44	0.0440	-2.25
Nkd1	7.5E-06	5.9E-06	1.26	0.5436	1.26
Nlk	8.9E-03	6.6E-03	1.36	0.4715	1.36
Pitx2	4.3E-02	2.7E-02	1.57	0.2367	1.57
Porcn	2.4E-02	1.8E-03	13.66	0.0000	13.66
Ppp2ca	4.7E-01	4.2E-01	1.11	0.5838	1.11
Ppp2r1a	2.1E-01	1.9E-01	1.07	0.6229	1.07
Ppp2r5d	5.1E-02	4.0E-02	1.27	0.5265	1.27
Pygo1	9.1E-04	2.3E-04	4.03	0.0006	4.03
Rhou	2.4E-02	2.0E-02	1.17	0.3873	1.17
Senp2	2.3E-02	2.2E-02	1.04	0.9028	1.04
Sfrp1	3.3E-03	1.3E-03	2.65	0.0160	2.65
Sfrp2	2.1E-03	3.3E-04	6.39	0.0000	6.39
Sfrp4	6.1E-04	7.0E-04	0.86	0.3131	-1.16
Slc9a3r1	2.1E-02	2.9E-02	0.75	0.3117	-1.34
Sox17	7.4E-06	5.9E-06	1.25	0.5528	1.25
T	7.4E-06	6.9E-06	1.06	0.8977	1.06
Tcf3	1.5E-02	6.1E-03	2.54	0.0279	2.54
Tcf7	2.7E-02	2.6E-02	1.07	0.6984	1.07
Tle1	1.6E-02	1.4E-02	1.14	0.4921	1.14
Tle2	3.8E-04	7.9E-05	4.83	0.0110	4.83
Wif1	7.5E-06	5.9E-06	1.27	0.5111	1.27

Wisp1	1.4E-01	8.4E-02	1.62	0.0729	1.62
Wnt1	1.2E-05	1.7E-05	0.71	0.6308	-1.41
Wnt10a	4.3E-03	5.9E-04	7.26	0.0013	7.26
Wnt11	6.7E-06	7.0E-06	0.95	0.9235	-1.05
Wnt16	9.2E-05	1.3E-04	0.73	0.5399	-1.37
Wnt2	6.7E-06	7.1E-06	0.93	0.8920	-1.07
Wnt2b	1.3E-03	5.8E-04	2.29	0.0003	2.29
Wnt3	1.0E-05	6.1E-06	1.66	0.3371	1.66
Wnt3a	6.7E-06	5.9E-06	1.12	0.7580	1.12
Wnt4	5.1E-04	2.3E-04	2.24	0.0145	2.24
Wnt5a	1.2E-05	6.8E-06	1.79	0.0493	1.79
Wnt5b	3.8E-04	5.3E-04	0.72	0.3852	-1.38
Wnt6	2.9E-04	8.5E-05	3.38	0.0437	3.38
Wnt7a	6.7E-06	8.0E-06	0.83	0.7030	-1.20
Wnt7b	3.2E-04	4.3E-04	0.74	0.6052	-1.36
Wnt8a	6.7E-06	5.9E-06	1.12	0.7580	1.12
Wnt8b	8.1E-06	6.3E-06	1.29	0.3876	1.29
Wnt9a	3.4E-03	3.6E-04	9.37	0.0018	9.37
