# Supplementary material for: Canonical Wnt signaling is involved in switching from cell proliferation to myogenic differentiation of mouse myoblast cells
Source: J Mol Signal. 2011 Oct 5;6:12. doi: 10.1186/1750-2187-6-12 (PMC3198762; doi:10.1186/1750-2187-6-12)
Supplement: Additional file 3 — Real-time PCR results (Day 4 vs control). Array results of real-time PCR analysis. n = 3 for the Day 4 group and n = 4 for the control group. [file 1750-2187-6-12-S3.PDF]

Real time RT-PCR results (Day 4 vs control)

Symbol	$2^{-\Delta C_t}$		Fold Difference	T-TEST	Fold Up- or Down-Regulation
	Day 4	Control Sample	Day 4 /Control Sample	p value	Day 4 /Control Sample
Aes	1.7E-01	9.8E-02	1.75	0.0806	1.75
Apc	4.4E-02	2.3E-02	1.86	0.0767	1.86
Axin1	1.8E-02	8.8E-03	2.05	0.1112	2.05
Bcl9	3.6E-03	2.0E-03	1.79	0.0057	1.79
Btrc	6.6E-03	3.9E-03	1.69	0.0185	1.69
Ctnnbip1	1.1E-02	7.0E-03	1.62	0.1955	1.62
Ccnd1	5.7E-02	1.2E-01	0.47	0.0256	-2.13
Ccnd2	9.6E-02	9.1E-02	1.05	0.8139	1.05
Ccnd3	4.4E-01	8.9E-02	4.95	0.0003	4.95
Csnk1a1	2.4E-01	1.0E-01	2.30	0.0009	2.30
Csnk1d	1.2E-01	8.1E-02	1.44	0.1286	1.44
Csnk2a1	2.4E-01	1.3E-01	1.91	0.0015	1.91
Ctbp1	5.3E-02	2.3E-02	2.31	0.0968	2.31
Ctbp2	7.0E-02	7.1E-02	0.98	0.9479	-1.02
Ctnnb1	2.5E-01	1.1E-01	2.21	0.0316	2.21
Daam1	6.5E-02	3.8E-02	1.72	0.0666	1.72
Dixdc1	2.0E-03	1.5E-03	1.31	0.1073	1.31
Dkk1	7.9E-06	5.9E-06	1.32	0.6099	1.32
Dvl1	2.3E-02	8.8E-03	2.59	0.0215	2.59
Dvl2	1.6E-02	6.9E-03	2.30	0.0630	2.30
Ep300	3.1E-02	1.6E-02	1.94	0.0002	1.94
Fbxw11	4.0E-02	2.7E-02	1.48	0.2725	1.48
Fbxw2	8.7E-02	4.9E-02	1.77	0.0153	1.77
Fbxw4	1.1E-02	4.9E-03	2.25	0.1008	2.25
Fgf4	7.9E-06	5.9E-06	1.32	0.6099	1.32
Fosl1	1.4E-02	8.5E-02	0.17	0.0003	-6.04
Foxn1	9.0E-04	6.9E-04	1.30	0.1322	1.30
Frat1	1.3E-04	3.8E-05	3.35	0.0079	3.35
Frzb	1.7E-05	2.6E-05	0.67	0.4010	-1.49
Fshb	1.6E-05	2.4E-05	0.64	0.6326	-1.56

Fzd1	5.2E-02	1.2E-02	4.21	0.0013	4.21
Fzd2	7.5E-03	2.1E-03	3.50	0.0080	3.50
Fzd3	1.2E-02	7.9E-03	1.53	0.0587	1.53
Fzd4	2.1E-03	1.0E-03	2.02	0.0517	2.02
Fzd5	3.0E-02	6.2E-03	4.78	0.0007	4.78
Fzd6	1.1E-02	8.6E-03	1.24	0.3403	1.24
Fzd7	3.1E-03	1.1E-03	2.70	0.2126	2.70
Fzd8	8.2E-05	2.0E-05	4.13	0.0374	4.13
Gsk3b	1.4E-02	5.7E-03	2.47	0.0034	2.47
Jun	5.7E-02	5.2E-02	1.08	0.7931	1.08
Kremen1	5.6E-02	4.0E-02	1.40	0.3010	1.40
Lef1	3.8E-03	2.5E-03	1.49	0.2132	1.49
Lrp5	2.3E-02	1.1E-02	2.15	0.0846	2.15
Lrp6	4.3E-02	1.9E-02	2.22	0.0013	2.22
Myc	2.4E-02	4.0E-02	0.59	0.1555	-1.70
Nkd1	8.0E-06	5.9E-06	1.34	0.5885	1.34
Nlk	7.9E-03	6.6E-03	1.21	0.7057	1.21
Pitx2	5.1E-02	2.7E-02	1.86	0.1465	1.86
Porcn	3.6E-02	1.8E-03	19.96	0.0001	19.96
Ppp2ca	5.9E-01	4.2E-01	1.41	0.0839	1.41
Ppp2r1a	2.3E-01	1.9E-01	1.20	0.2730	1.20
Ppp2r5d	4.5E-02	4.0E-02	1.12	0.7739	1.12
Pygo1	1.6E-03	2.3E-04	7.10	0.0001	7.10
Rhou	2.7E-02	2.0E-02	1.31	0.2943	1.31
Senp2	2.6E-02	2.2E-02	1.20	0.5651	1.20
Sfrp1	3.9E-03	1.3E-03	3.07	0.0106	3.07
Sfrp2	2.4E-03	3.3E-04	7.02	0.0000	7.02
Sfrp4	7.3E-04	7.0E-04	1.04	0.7686	1.04
Slc9a3r1	2.0E-02	2.9E-02	0.70	0.2319	-1.44
Sox17	1.1E-05	5.9E-06	1.87	0.1979	1.87
T	8.4E-06	6.9E-06	1.21	0.7603	1.21
Tcf3	1.7E-02	6.1E-03	2.74	0.0191	2.74
Tcf7	3.5E-02	2.6E-02	1.36	0.0914	1.36
Tle1	1.6E-02	1.4E-02	1.18	0.4120	1.18
Tle2	3.5E-04	7.9E-05	4.41	0.0159	4.41
Wif1	9.3E-06	5.9E-06	1.56	0.3854	1.56

Wisp1	2.1E-01	8.4E-02	2.47	0.0087	2.47
Wnt1	2.0E-05	1.7E-05	1.13	0.8369	1.13
Wnt10a	4.6E-03	5.9E-04	7.84	0.0030	7.84
Wnt11	7.9E-06	7.0E-06	1.13	0.8501	1.13
Wnt16	1.1E-04	1.3E-04	0.87	0.7702	-1.15
Wnt2	7.9E-06	7.1E-06	1.10	0.8810	1.10
Wnt2b	8.8E-04	5.8E-04	1.51	0.0755	1.51
Wnt3	7.9E-06	6.1E-06	1.29	0.6474	1.29
Wnt3a	9.4E-06	5.9E-06	1.58	0.3965	1.58
Wnt4	3.1E-04	2.3E-04	1.38	0.3619	1.38
Wnt5a	7.9E-06	6.8E-06	1.17	0.7470	1.17
Wnt5b	3.8E-04	5.3E-04	0.72	0.3695	-1.39
Wnt6	3.6E-04	8.5E-05	4.29	0.0032	4.29
Wnt7a	8.0E-06	8.0E-06	1.00	0.9952	1.00
Wnt7b	2.2E-04	4.3E-04	0.52	0.3093	-1.93
Wnt8a	7.9E-06	5.9E-06	1.32	0.6099	1.32
Wnt8b	1.2E-05	6.3E-06	1.92	0.1935	1.92
Wnt9a	3.7E-03	3.6E-04	10.20	0.0019	10.20
