# Supplementary figures and images for: Canonical Wnt signaling is involved in switching from cell proliferation to myogenic differentiation of mouse myoblast cells
Source: J Mol Signal. 2011 Oct 5;6:12. doi: 10.1186/1750-2187-6-12 (PMC3198762; doi:10.1186/1750-2187-6-12)

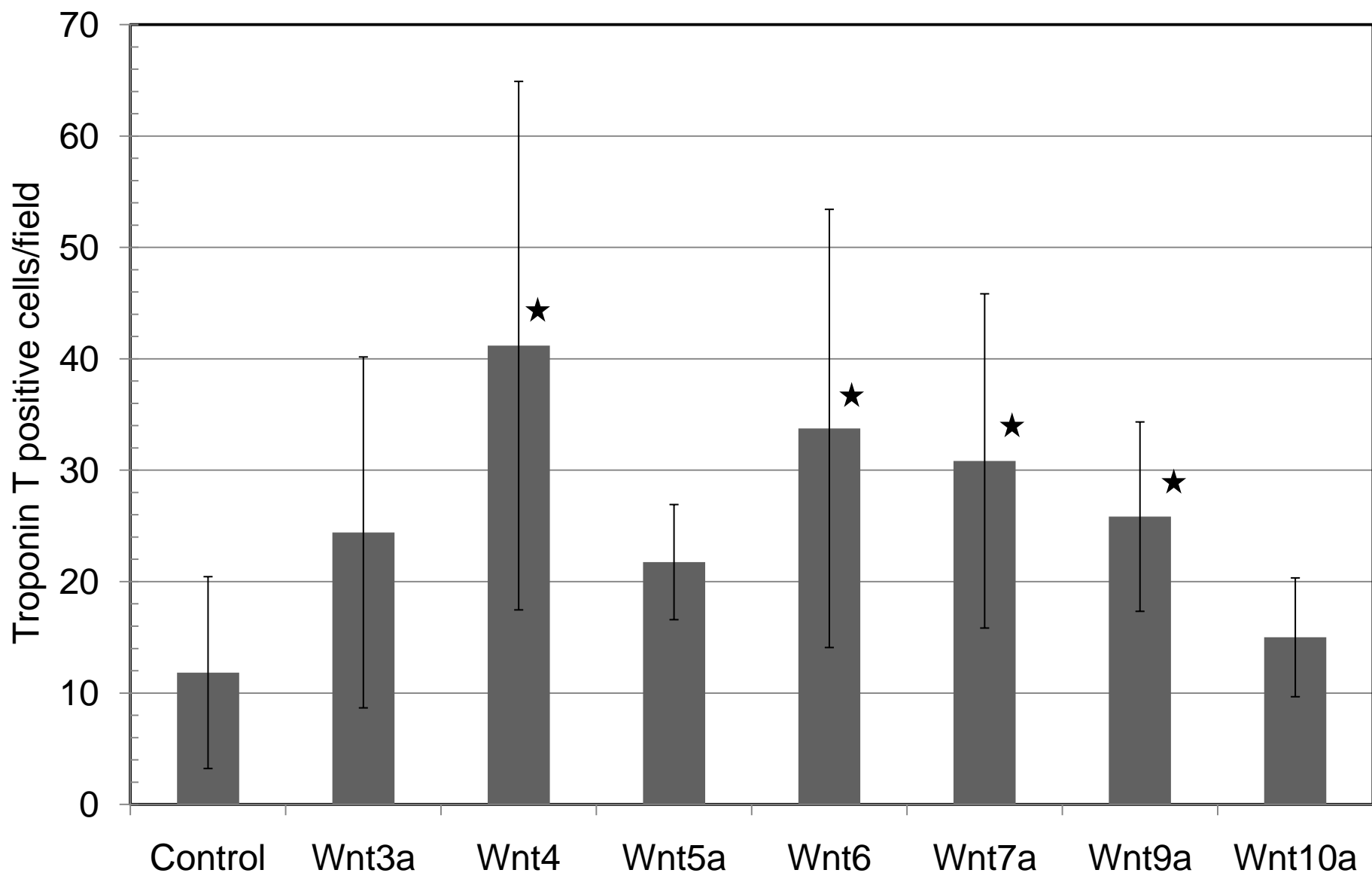

Supplement: Additional file 4 — Effect of Wnt expression on cell differentiation. C2C12 cells were transfected with Wnt cDNAs in pcDNA3.2 and cultured in proliferation medium for 2 days. Cells were fixed, troponin T immunostained and positive cell nuclei were counted. *P < 0.03 vs. control. [file 1750-2187-6-12-S4.PDF]
